# Supplementary material for: Economic burden of the persistent morbidity of nodding syndrome on caregivers in affected households in Northern Uganda
Source: PLoS One. 2020 Sep 29;15(9):e0238643. doi: 10.1371/journal.pone.0238643 (PMC7523991; doi:10.1371/journal.pone.0238643)
Supplement: S1 File — (DOCX) [file pone.0238643.s001.docx]

## **Appendix C – 1 Questionnaire**

| **Research Questionnaire** |
| --- |
|  |
| **General Instructions** |

The questionnaire is divided into the following sections below

|  |  |
| --- | --- |
| Section 1: | Household rooster and Household characteristic |
|  |  |
| Section 2: | Respondent’s work or employment Characteristics |
|  |  |
| Section 3: | Direct medical and non-medical costs |
|  |  |
| Section 4: | Characteristics of the informal care situation |
|  |  |
| Section 5: | Subjective burden, health and well-being and how respondent cope. |
|  |  |
|  |  |
| Section 6: | Seizure frequency and severity |
|  |  |

|  |  |  | d | d |  | m | m |  | y | | y | y | | y |  |  |  | | | |
| --- | --- | --- | --- | --- | --- | --- | --- | --- | --- | --- | --- | --- | --- | --- | --- | --- | --- | --- | --- | --- |
|  |  |  |  |  |  |  |  |  |  | |  |  | |  |  |  |  |  |  |  |
|  | Interview Date |  |  |  |  |  |  |  |  | |  |  | |  |  |  | Questionnaire Number |  |  |  |
|  |  |  |  |  |  |  |  |  |  | |  |  | |  |  |  |  |  |  |  |
|  |  |  |  |  |  |  |  |  |  | |  |  | |  |  |  |  | | | |
|  |  |  |  |  |  |  |  |  |  | |  |  | |  |  |  |  |  |  |  |
|  | Interviewer code …………… | | | | | |  | |  |  | | |  |  |  |  | Household ID |  |  |  |
|  |  |  |  |  |  |  |  |  |  | |  |  | |  |  |  |  |  |  |  |

| **SECTION 1 – Household Roster**  **I am now going to ask you questions about each usual members of the household.** | | | | | | | | | |
| --- | --- | --- | --- | --- | --- | --- | --- | --- | --- |
| No. | 1 | 2 | 3 | 4 | 5 | 6 | 7 | 8 | 9 |
|  | First name | Sex | Age (years) | Marital status | Relationship to household head | Highest level of education attained | Religious affiliation | Patient of NS /Epilepsy | Patient caregiver? |
|  |  | Male……1  Female…2 |  | Married 1  Living with a partner 2  Divorced/separated 3  Widow/ widower 4  Never Married 5  No response...............-99 | Head 1  Wife/Husband 2  Son/Daughter 3  Son/Daughter-in-law 4  Grandchild 5  Parent 6  Parent in law 7  Brother/Sister 8  Nephew/Niece/Cousin 9  Other 10  Don’t know................-88  No response................-99 | No education 0  Primary 1  ‘O’ Level 2  ‘A’ Level 3  Tertiary 4  University 5  Vocation education 6  No response -99 | No religion 1  Anglican 2  Catholic 3  Muslim 4  Seventh Day Adventist ……… 5  Pentecostal / Born Again /Evangelical ……..6  Traditional Religion……7  Other denominations……8 | Yes…….1  No……..2 | Yes…….1  No……..2 |
| 1 |  |  |  |  |  |  |  |  |  |
| 2 |  |  |  |  |  |  |  |  |  |
| 3 |  |  |  |  |  |  |  |  |  |
| 4 |  |  |  |  |  |  |  |  |  |
| 6 |  |  |  |  |  |  |  |  |  |
| 7 |  |  |  |  |  |  |  |  |  |
| 8 |  |  |  |  |  |  |  |  |  |
| **Are there other family members (say for example who sleep) somewhere else but are members of this household?** | | | | | | | | Yes…….1  No…….2 | List below |
| 9 |  |  |  |  |  |  |  |  |  |
| 10 |  |  |  |  |  |  |  |  |  |

| **Qn 10** | | **Household Characteristics** | | |
| --- | --- | --- | --- | --- |
| **Please observe the floors, roof and exterior walls and tick accordingly and if need be, ask questions about household characteristics** | | | | |
|  |  | | Natural Floor |  |
|  |  | | Earth/Sand 1 |  |
|  |  | | Dung 2 |  |
|  |  | | Rudimentary Floor |  |
|  |  | | Wood Planks 3 |  |
|  |  | | Palm/Bamboo 4 |  |
| a): | Main material of the floor | | Finished Floor  Parquet or polished wood 5 |  |
|  | **OBSERVE** | | Vinyl or Asphalt strips 6 |  |
|  |  | | Ceramic Tiles 7 |  |
|  |  | | Cement 8 |  |
|  |  | | Carpet 9 |  |
|  |  | | Other 10 |  |
|  |  | | No response ...-99 |  |
|  |  | | No Roof 10 |  |
|  |  | | Natural Roofing |  |
|  |  | | Grass/Thatch/Makuti 11 |  |
|  |  | | Dung/Mud 12 |  |
|  |  | | Rudimentary Roofing |  |
| b): | Main material of the roof | | Corrugated Iron (Mabati) 21  Tin Cans 22 |  |
|  | **OBSERVE** | | Finished Roofing |  |
|  |  | | Asbestos Sheet 31 |  |
|  |  | | Cement 32 |  |
|  |  | | Tiles 33 |  |
|  |  | | Other 96 |  |
|  |  | | No response ...-99 |  |
|  |  | | No Walls 11 |  |
|  |  | | Natural Walls |  |
|  |  | | Cane/Palm/Trunks 12 |  |
|  |  | | Dirt 13 |  |
|  |  | | Rudimentary Walls |  |
|  |  | | Bamboo with Mud 21 |  |
|  |  | | Stone with Mud 22 |  |
|  |  | | Uncovered Adobe 23 |  |
|  |  | | Plywood 24 |  |
| c): | Main material of the exterior walls | | Cardboard 25  Reused Wood 26 |  |
|  | **OBSERVE** | | Finished Walls |  |
|  |  | | Cement 31 |  |
|  |  | | Stone with Lime/Cement 32 |  |
|  |  | | Bricks 33 |  |
|  |  | | Cement Blocks 34 |  |
|  |  | | Covered Adobe 35 |  |
|  |  | | Wood Planks /Shingles 36 |  |
|  |  | | Other 96 |  |
|  |  | | No response ...-99 |  |

| d): | **What is the main source of drinking water for members of your household?**  Piped Water  Piped into dwelling/indoor..............................  Pipe to yard/plot..............................................  Publictap/standpipe..................................................  Tube well or borehole..............................................  Dug Well  Protected Well.................................................  Unprotected Well............................................  Water from Spring  Protected Spring.............................................  Unprotected Spring.......................................  Rainwater.................................................................  Tanker Truck...........................................................  Cart with Small Tank...............................................  Surface water (River / Dam / Lake / Pond /  Stream / Canal / Irrigation Channel) .......................  BottledWater............................................................  SachetWater.............................................................  No response.............................................................. | ..........................................................................1  ..........................................................................2  ..........................................................................3  ..........................................................................4  ..........................................................................5  ..........................................................................6  ..........................................................................7  ..........................................................................8  ..........................................................................9  ........................................................................10  ........................................................................11  ........................................................................12  ........................................................................13  ........................................................................14  ....................................................................... -99 |  |
| --- | --- | --- | --- |
| e): | **What is the main source of water used by your household for other purposes such as cooking and handwashing?**  Piped Water  Piped into dwelling/indoor ......................................  Pipe to yard/plot.......................................................  Public tap/standpipe.................................................  Tube well or borehole..............................................  Dug Well  Protected Well .........................................................  Unprotected Well.....................................................  Water from Spring.  Protected Spring ......................................................  Rainwater.................................................................  Tanker Truck ...........................................................  Cart with Small Tank...............................................  Surface water (River / Dam / Lake / Pond /  Stream / Canal / Irrigation Channel) .......................  Bottled Water...........................................................  Sachet Water............................................................  No response ............................................................. | .......................................................................... 1  .......................................................................... 2  .......................................................................... 3  .......................................................................... 4  .......................................................................... 5  .......................................................................... 6  .......................................................................... 7  .......................................................................... 8  .......................................................................... 9  ........................................................................ 10  ........................................................................ 11  ........................................................................ 12  ........................................................................ 13  ....................................................................... -99 |  |

| f): | **Do members of your household use any of the following toilet facilities?**  Flush/pour flush toilets connected to:  Piped sewer system.........................................  Septic tank......................................................  Elsewhere.......................................................  Unknown / Not sure / Don’t know..................  Ventilated improved pit latrine................................  Pit latrine with slab..................................................  Pitlatrinewithoutslab.............................................  Composting toilet....................................................  Buckettoilet.............................................................  Hanging toilet /Hanging latrine...............................  Other (please explain): ............................................  No facility/bush/field...............................................  No response.............................................................. | .....................................................................  .....................................................................  .....................................................................  ....................................................................  .....................................................................  .....................................................................  .....................................................................  .....................................................................  .....................................................................  .....................................................................  .....................................................................  .......................................................................................................................................... | Yes  1  1  1  1  1  1  1  1  1  1  1  1  -99 | No  0  0  0  0  0  0  0  0  0  0  0  0  0 |  |
| --- | --- | --- | --- | --- | --- |

| g): | **What type of fuel does your household mainly use for cooking?**  Electricity ............................ ...............................1  Cylinder gas ........................................................2  Biogas kerosene....................................................3  Charcoal...............................................................4  Wood ...................................................................5  Straw/srubs/grass.................................................6  Agricultural crop .................................................7  Animal dung ........................................................8  No food cooked in the household .......................9  Others: Specify__________________________  No response ......................................................-99 | h): | **Does your household have?**  Electricity .........................................  Radio ..............................................  Television .......................................  Non-mobile phone .........................  Refrigerator ...... .............................  Computer .......................................  Table ........ .....................................  Chair ........... ...................................  Sofa set ....... ...................................  Bed ............................. ................... Cupboard .... ...................................  No response .... ............................... | Yes  1  1  1  1  1  1  1  1  1  1  1  -99 | No  0  0  0  0  0  0  0  0  0  0  0 |  |
| --- | --- | --- | --- | --- | --- | --- |

| i): | **Does any member of your household have?**  Watch ...........................................  Mobile phone................................  Bicycle .........................................  Motor bicycle .............................  Animal drawn cart .......................  Car /truck .....................................  Boat with motor ....... ...................  Boat without motor ......................  No response ....... .......................... | Yes  1  1  1  1  1  1  1  1  -99 | No  0  0  0  0  0  0  0  0 | j): | **How many of the following animal does your household own?** | | |  |
| --- | --- | --- | --- | --- | --- | --- | --- | --- |
|  |  |  |  |  | Local cattle? .......................................... |  |  |  |
|  |  |  |  |  | Exotic/cross-breed cattle? ....................... |  |  |  |
|  |  |  |  |  | Horses, donkeys, or mules? ..................... |  |  |  |
|  |  |  |  |  | Goats? .................................................. |  |  |  |
|  |  |  |  |  | Sheep? ................................................... |  |  |  |
|  |  |  |  |  | Chickens or other poultry? ..................... |  |  |  |
|  |  |  |  |  | Pigs? ....................................................... |  |  |  |
|  |  |  |  |  |  |  |  |  |

| **Section 2:** | | **Respondent’s work or employment Characteristics** |  | |  |
| --- | --- | --- | --- | --- | --- |
|  | |  |  | |  |
| Q11 | As you know, some people take up jobs for which they are paid in cash or kind. Others sell things, have a small business or work on the family farm or in the family business. In the past seven days or 12 months, have you done any of these things or any other work? | |  | |  |
|  | Yes | | 1 | → Q14 | |
|  | No | | 2 |  | |
| Q12 | Although you did not work in the past seven days or 12 months, did you have such work, from which you were temporarily absent for leave, illness, vacation, or any other such reason? | |  | |  |
|  | Yes | | 1 | | → Q14 |
|  | No | | 2 | |  |
| Q13 | In the past seven days or 12 months, did you help without being paid in any kind of business run by your household, or volunteering elsewhere even if it was only for one hour? | |  | |  |
|  | Yes | | 1 | |  |
|  | No | | 2 | |  |
| Q14 | What is your occupation? That is, what kind of work do you mainly do? | |  | |  |
|  | __________________________________________________________ | |  | |  |
| Q15 | Are you paid in cash or kind for this work or are you not paid at all? | |  | |  |
|  | Cash only | | 1 | |  |
|  | Cash and Kind | | 2 | |  |
|  | In Kind only | | 3 | |  |
|  | Not Paid | | 4 | |  |
| Q16a | What is your income level? | |  | |  |
|  | Specify amount ________________________________________________ | | 1 | |  |
|  | Less than UGX 50,000 per month ……………………………………...……………………………. | | 2 | |  |
|  | Between UGX (50,000 – 99,999) per month ………………………….…………………………….. | | 3 | |  |
|  | Between UGX (100,000 – 199,999) per month ………………………………………………. | | 4 | |  |
|  | Between UGX (200,000 – 2 99,999) per month. ………………………………………………. | | 5 | |  |
|  | Between UGX (300,000 – 399,999) per month. ………………………………………………. | | 6 | |  |
|  | Between UGX (400,000 – 499,999) per month ………………………………………………. | | 7 | |  |
|  | Between UGX (500,000 – 599,999) per month. ………………………………………………. | | 8 | |  |
|  | Between UGX (600,000 – 699,999) per month. ………………………………………………. | | 9 | |  |
|  | Between UGX (700,000 – 799,999) per month ………………………………………………. | | 10 | |  |
|  | Between UGX (800,000 – 899,999) per month. ………………………………………………. | | 11 | |  |
|  | Between UGX (900,000 – 999,999) per month. ………………………………………………. | | 12 | |  |
|  | From UGX 1,000,000 and above per month …………………………………………………. | | 13 | |  |
|  | I do not know. ……………………………………………………………… | | 98 | |  |
| Q16b | If working without pay or not working at all, suppose you get a job that is in line with your education, professional qualifications, skills, and experiences, how much money would you minimally expect to receive? | |  | |  |
|  | _________________________________________________________in a month?” | |  | |  |
| Q17a | In the past seven days or 12 months, did you have any secondary job/activity from which you or your households obtain any income in cash or in kind? | |  | |  |
|  | Yes ………………………………………………………………………………………………… | | 1 | |  |
|  | No ………………………………………………………………………………………………. | | 2 | |  |

| Q17b | What kind of work do you usually do in this secondary job/activity? (*Examples: street seller, subsistence farmer, primary school teacher, registered nurse, domestic worker, truck driver)* |  |  |
| --- | --- | --- | --- |
|  | _______________________________________ |  |  |
| Q17c | If paid in cash, specify amount  ________________________________________ |  |  |
| Q17d | If paid in kind, specify in kind benefits  __________________________________ |  |  |
| Q18a | Do you usually work throughout the year, or do you work seasonally, or only once in a while? |  |  |
|  | throughout the year . . . . . . . . . . . . . . .. | 1 |  |
|  | seasonally/part of the year . . . . . . . . .. | 2 |  |
|  | once in a while . . . . . . . . . . . . . . . . . . . . . . . . | 3 |  |
| Q18b | If working seasonally/ part of the year or once in a while, what is the reason? |  |  |
|  | _______________________________________ |  |  |
| Q19 | Do you own any agricultural or non-agricultural land either alone or jointly with someone else? |  |  |
|  | Alone only ……………………………………………………………………………………… | 1 |  |
|  | Jointly only ………………………………………………………………………………………… | 2 |  |
|  | Both alone and jointly …………………………………………………………………………… | 3 |  |
|  | Does not own …………………………………………………………………………………… | 4 |  |
| Q20a | Has your partner (husband/wife) done any work in the past seven days or 12 months? |  |  |
|  | Yes ………………………………………………………………………………………………. | 1 |  |
|  | No ………………………………………………………………………………………………. | 2 |  |
| Q20b | What is his/her occupation? That is, what kind of work does he/she mainly do? |  |  |
|  | ___________________________________________________________________ |  |  |
| Q20c | Would you say that the money that you earn is more than what your (husband/partner) earns, less than what he/she earns, or about the same? |  |  |
|  | More than him/her . . . . . . . . . . . . . . . . . . . . . . . . | 1 |  |
|  | Less than him/her . . . . . . . . . . . . . . . . . . . . . . . . | 2 |  |
|  | About the same . . . . . . . . . . . . . . . . . . . . . . . . | 3 |  |
|  | Husband/Wife has no earnings | 4 |  |
|  | Don't know . . . | 98 |  |
|  | How would you describe your household's overall financial situation? |  |  |
|  | Well off ………………………………………………………………………………………… | 1 |  |
| Q21 | Fairly well off …………………………………………………………………………………… | 2 |  |
|  | Average ………………………………………………………………………………………… | 3 |  |
|  | Fairly poor …………………………………………………………………………………… | 4 |  |
|  | Poor ………………………………………………………………………………………………. | 5 |  |

| **Section 3:** | | **Direct medical and non-medical costs** |  |  |
| --- | --- | --- | --- | --- |
|  |  | |  |  |
| Q22 | Where do you normally seek treatment for the child / children with Nodding syndrome? | |  |  |
|  | Government Hospital ……………………………………………………………………… | | 1 |  |
|  | Government Health Center ……………………………………………………………………… | | 2 |  |
|  | Mobile Clinic ……………………………………………………………………… | | 3 |  |
|  | Private Hospital/Clinic ……………………………………………………………………… | | 4 |  |
|  | Private Doctor ……………………………………………………………………… | | 5 |  |
|  | Pharmacy/Drug Shop ……………………………………………………………………… | | 6 |  |
|  | Traditional practitioner ……………………………………………………………………… | | 7 |  |
|  | Market ……………………………………………………………………… | | 8 |  |
|  | Other (please specify): _______________________________________ .. | | 9 |  |
| Q23 | What was/were the most important reason/s for choosing this provider?  *(Please circle all that respondent mentions)* | |  |  |
|  | Good reputation | | 1 |  |
|  | Inexpensive | | 2 |  |
|  | Good personal experience | | 3 |  |
|  | Qualification of staff | | 4 |  |
|  | Availability of drugs | | 5 |  |
|  | Relative/Friend works here | | 6 |  |
|  | Other (please specify): _______________________________________ | | 7 |  |
| Q24 | How far is this provider from your home?  _______________________________________ | |  |  |
| Q25 | How do you travel to this provider? *(Please circle all that respondent mentions)* | |  |  |
|  | Private vehicle. …………………………………………………………… | | 1 |  |
|  | Public transportation (taxi, bus) ……………………………………… | | 2 |  |
|  | Ambulance or emergency vehicle …………………………………… | | 3 |  |
|  | Motor bike …………………………………………………………………… | | 4 |  |
|  | Bicycle …………………………………………………………...…………… | | 5 |  |
|  | Walked ………………………………………………………………………. | | 6 |  |
|  | Other (please specify): _______________________________________ | | 7 |  |
|  | How long does the journey take to go from your home to this provider? | |  |  |
| Q26 | ___________________ minutes or ___________________ hours …… | | 1 |  |
|  | I do not know …………………………………………………………… | | 98 |  |
| Q27 | Considering the last visit, how much in total did you pay for transport for the journey to and back from this provider _______________________________________amount | |  |  |
| Q28 | Who paid for this? [ *Please circle all that respondent mentions]* | |  |  |
|  | Respondent. ………………………………………………………………………………………. | | 1 |  |
|  | Spouse or partner. ………………………………………………………..…………………. | | 2 |  |
|  | Son / daughter …………………………………………………………………………………. | | 3 |  |
|  | Relative……………………………………………………….………………….………………… | | 4 |  |
|  | Non - family member …………………………………………………………………………. | | 5 |  |
|  | It was free …………………………………………………………...………...…………………. | | 6 |  |
|  | Other (please specify): _______________________________________ ... | | 7 |  |
|  | I do not know ………………………………………………………………………………. | | 98 |  |

| Q29 | Did you have to spend on the following? | | | | | | | | |
| --- | --- | --- | --- | --- | --- | --- | --- | --- | --- |
|  |  | | | If yes, approx. how much? | | | | | |
| i | Private accommodation | ○ | Yes | | → |  | | Amount | |
|  |  | ○ | No | |  |  | | | |
|  |  | ○ | n/a | |  |  | | | |
| ii | Feeding (food and drinks) | ○ | Yes | | → |  | | Amount | |
|  |  | ○ | No | |  |  | | | |
|  |  | ○ | n/a | |  |  | | | |
|  |  | | | | | | | | |
|  |  | | | | | | | | |
| Q30a | Did you have to spend on the following? | | | | | | | | |
|  |  | | | If yes, approx. how much? | | | | | |
| i | Registration (getting a booklet, etc.) | ○ | Yes | | → |  | | Amount | |
|  |  | ○ | No | |  |  | | | |
|  |  | ○ | n/a | |  |  | | | |
| ii | Consultations | ○ | Yes | | → |  | | Amount | |
|  |  | ○ | No | |  |  | | | |
|  |  | ○ | n/a | |  |  | | | |
| iii | Tests (laboratory and any other) | ○ | Yes | | → |  | | Amount | |
|  |  | ○ | No | |  |  | | | |
|  |  | ○ | n/a | |  |  | | | |
| iv | Medications (drugs) | ○ | Yes | | → |  | | Amount | |
|  |  | ○ | No | |  |  | | | |
|  |  | ○ | n/a | |  |  | | | |
| v | Hospital stays (hospitalization) | ○ | Yes | | → |  | | Amount | |
|  |  | ○ | No | |  |  | | | |
|  |  | ○ | n/a | |  |  | | | |
| vii | Other (please specify):  ____________________________________ | ○ | Yes | | → |  | | Amount | |
|  |  | ○ | No | |  |  | | | |
|  |  | ○ | n/a | |  |  | | | |
|  |  |  |  | |  |  | | | |
| Q30b | For the expenditure in question 30 above, how you make the payment | | | | | |  | |  |
|  | Directly out of pocket ……………………………………………………………………….. | | | | | | 1 | |  |
|  | Community-Based Initiative/Savings ………………………………………………………… | | | | | | 2 | |  |
|  | Health insurance through employer ………………………………………………………… | | | | | | 3 | |  |
|  | Social Security ……………………………………………………………………………… | | | | | | 4 | |  |
|  | Other privately purchased health insurance …………………………………………………… | | | | | | 5 | |  |
|  | Commercial health insurance ………………………………………………………………. | | | | | | 6 | |  |
|  | Other (please specify): _______________________________________ | | | | | | 7 | |  |

| Q31a | Did you receive all medicines at this facility, or you had to get others from private pharmacy? |  | |  |
| --- | --- | --- | --- | --- |
|  | Receive all medicine at current facility | 1 | |  |
|  | Buy other drugs from pharmacy ……………………………………… | 2 | | → Q14a |
| Q31b | If other drugs are got from the pharmacy, how much do you pay on average? | |  |  |
|  | _______________________________________ | |  |  |
| Q32a | Yes ………………………………………………………………………… | | 1 |  |
|  | No …………………………………………………………………………. | | 2 |  |
| Q32b | If yes, specify item and amount below | |  |  |
|  | Item ________________________________ amount __________________________ | | | |
|  | Item ________________________________ amount __________________________ | | | |
|  | Item ________________________________ amount __________________________ | | | |
| Q33 | What were the other diagnoses? (check medical records; booklet if available)  **__________________________** | | | |

| Section 4 | | Characteristics of the informal care situation | | |
| --- | --- | --- | --- | --- |
|  | | | | |
| With the following questions we would like to get an impression of the informal care you provide. | | | | |
| Q34 | Do you provide care or support on a voluntarily basis to a family member, friend or other acquaintance who needs help due to his/her health condition (epilepsy/nodding syndrome)? | |  |  |
|  | Yes ………………………………………………………………………… | | 1 |  |
|  | No …………………………………………………………………………. | | 2 |  |
|  | How long have you been providing informal care to her/him? | |  |  |
| Q35 | Less than a month: ________________________weeks ……………… | | 1 |  |
|  | Less than a year:___________________________months …………… | | 2 |  |
|  | More than a year: __________________________years ……………… | | 3 |  |
| Q36 | On how many days per week do you usually provide care to her/him? | |  |  |
|  | 1 days ……………………………………………………………………….. | | 1 |  |
|  | 2 days …………………………………………………………………….…. | | 2 |  |
|  | 3 days ……………………………………………………………………….. | | 3 |  |
|  | 4 days ……………………………………………………………………….. | | 4 |  |
|  | 5 days ……………………………………………………………………….. | | 5 |  |
|  | 6 days ……………………………………………………………………….. | | 6 |  |
|  | 7 days ……………………………………………………………………….. | | 7 |  |
|  | Can she/he be left alone? | |  |  |
| Q37 | No, she/he needs continuous surveillance……………………………….. | | 1 |  |
|  | Yes, but not for more than one hour……………………………………….. | | 2 |  |
|  | Yes, she/he can easily be left alone for several hours …….. | | 3 |  |
|  | Do you share a household with her/him? | |  |  |
| Q38 | No, I live ______________minutes of travel distance from her/him……...… | | 1 |  |
|  | Yes ………………………………………………………………………..……………. | | 2 |  |

|  | Does she/he live independently? |  |  |
| --- | --- | --- | --- |
| Q39 | Yes, and she/he lives alone. …………………………..………………….. | 1 |  |
|  | Yes, and she/he shares a household with at least one other person…… | 2 |  |
|  | Other: _______________________________________ …..……………..…… | 3 |  |
| Q40a | Do you sometimes take him or her to other places like a relative’s place or any other place where he/she can be looked after by other people? |  |  |
|  | No ………………………………………………………………………..…… | 1 |  |
|  | Yes, for____________________________ hours during the last week…...... | 2 |  |
| Q40b | Besides your care or support, does she/he also receive care from other informal caregivers? |  |  |
|  | No, I am the only informal caregiver ……………………..……………....... | 1 |  |
|  | Yes, from ____________________________ [number] other informal caregivers, in total for ___________________hours during the last week | 2 |  |
|  |  |  |  |
|  |  |  |  |

| \| Q41a \| \| Did you spend time during the last week on the following activities in her/his house? \| \| \| \| \| \| \| \| \| \| \| \| \| \| \| \| \| \| \| \| \| \| \| \| \| --- \| --- \| --- \| --- \| --- \| --- \| --- \| --- \| --- \| --- \| --- \| --- \| --- \| --- \| --- \| --- \| --- \| --- \| --- \| --- \| --- \| --- \| --- \| --- \| --- \| --- \| \|  \|  \| \| \| \| \|  \| \| \| \| \| \| \| \| \| \|  \|  \| \| \| \| \| \| \| \| \| \|  \|  \| \| \| \| \| Minutes per day \| \| \| \| \| \| \| \| \| \| \| Hours per week \| \| \| \| \| \| \| \| \| \| i \| Food and drink preparation, doing the dishes \| \| \| \| ○ \| \| Yes \| \| \| → \| \| \|  \| \| \| \| \| \| \| or \| \|  \| \| \|  \| \| ○ \| \| No \| \| \|  \| \| \|  \| \| \| \| \| \| \|  \| \|  \| \| \| \| \| ii \| Cleaning the house or compound \| \| \| \| ○ \| \| Yes \| \| \| → \| \| \|  \| \| \| \| \| \| \| or \| \|  \| \| \|  \| \| ○ \| \| No \| \| \|  \| \| \|  \| \| \| \| \| \| \|  \| \|  \| \| \| \| \| iii \| Washing, mending, ironing clothes \| \| \| \| ○ \| \| Yes \| \| \| → \| \| \|  \| \| \| \| \| \| \| or \| \|  \| \| \|  \| \| ○ \| \| No \| \| \|  \| \| \|  \| \| \| \| \| \| \|  \| \|  \| \| \| \| \| iv \| Taking care of and playing with your children? \| \| \| \| ○ \| \| Yes \| \| \| → \| \| \|  \| \| \| \| \| \| \| or \| \|  \| \| \|  \| \| ○ \| \| No \| \| \|  \| \| \|  \| \| \| \| \| \| \|  \| \|  \| \| \| \| \| v \| Shopping household supplies (incl. food) \| \| \| \| ○ \| \| Yes \| \| \| → \| \| \|  \| \| \| \| \| \| \| or \| \|  \| \| \|  \| \| ○ \| \| No \| \| \|  \| \| \|  \| \| \| \| \| \| \|  \| \|  \| \| \| \| \| vi \| Other tasks, Grinding, pounding, collection of Water and Fuel (e.g. firewood, charcoal) \| \| \| \| ○ \| \| Yes \| \| \| → \| \| \|  \| \| \| \| \| \| \| or \| \|  \| \| \|  \| \| ○ \| \| No \| \| \|  \| \| \|  \| \| \|  \|  \| \| \| \| \| \| \| \| \| \|  \|  \| \| \| \| \| \| \| \| \| \| \| \| \| \| \| \| \| \| \| \| \| \| \| \| \| \| Q41b \| \| Did you spend time during the last week assisting her/him with the activities below? \| \| \| \| \| \| \| \| \| \| \| \| \| \| \| \| \| \| \| \| \| \| \| \| \|  \|  \| \|  \| \| \|  \| \| \|  \| \| \|  \| \| \| \| \| \|  \| \| \|  \| \| \| \| \| \|  \|  \| \| \| \| \| Minutes per day \| \| \| \| \| \| \| \| \| \| \| Hours per week \| \| \| \| \| \| \| \| \| \| i \| Personal care (dressing/undressing, washing, combing, shaving)? \| \| \| ○ \| \| \| Yes \| \| \| \| → \| \| \|  \| \| \| \| \| or \| \| \| \| \|  \|  \| \| ○ \| \| \| No \| \| \| \|  \| \| \|  \| \| \| \| \|  \| \| \| \| \|  \| \| \| ii \| Going to the toilet? \| \| \| ○ \| \| \| Yes \| \| \| \| → \| \| \|  \| \| \| \| \| or \| \| \| \| \|  \|  \| \| ○ \| \| \| No \| \| \| \|  \| \| \|  \| \| \| \| \|  \| \| \| \| \|  \| \| \| iii \| Moving around the house? \| \| \| ○ \| \| \| Yes \| \| \| \| → \| \| \|  \| \| \| \| \| or \| \| \| \| \|  \|  \| \| ○ \| \| \| No \| \| \| \|  \| \| \|  \| \| \| \| \|  \| \| \| \| \|  \| \| \| iv \| Eating and drinking? \| \| \| ○ \| \| \| Yes \| \| \| \| → \| \| \|  \| \| \| \| \| or \| \| \| \| \|  \|  \| \| ○ \| \| \| No \|  \| \| \| \| \| \|  \| \|  \|  \| \| \| \| \| \| \| \| \| \|  \| \|  \| \| \| \| \| \| \| \| \| \| \| \| \| \| \| \| \| \| \| \| \| \| \| \| \|  \| \|  \| \| \| \| \| \| \| \| \| \| \| \| \| \| \| \| \| \| \| \| \| \| \| \| \| Q41c \| \| Did you spend time during the last week assisting her/him with the activities below? \| \| \| \| \| \| \| \| \| \| \| \| \| \| \| \| \| \| \| \| \| \| \| \| \|  \|  \| \|  \| \| \|  \| \| \|  \| \| \|  \| \| \| \| \| \|  \| \| \|  \| \| \| \| \| \|  \|  \| \| \| \| \| Minutes per day \| \| \| \| \| \| \| \| \| \|  \| Hours per week \| \| \| \| \| \| \| \| \| \| i \| Mobility outside the house (assistance with walking or wheelchair)? \| \| \| ○ \| \| \| Yes \| \| \| → \| \| \| \| \|  \| \| \| \| \| or \| \| \|  \| \|  \| \| ○ \| \| \| No \| \| \|  \| \| \| \| \|  \| \| \| \| \|  \| \| \|  \| \| \| \| ii \| Making trips and visiting family or friends? \| \| \| ○ \| \| \| Yes \| \| \| → \| \| \| \| \|  \| \| \| \| \| or \| \| \|  \| \|  \| \| ○ \| \| \| No \| \| \|  \| \| \| \| \|  \| \| \| \| \|  \| \| \|  \| \| \| \| iii \| Visiting a doctor or the hospital \| \| \| ○ \| \| \| Yes \| \| \| → \| \| \| \| \|  \| \| \| \| \| or \| \| \|  \| \|  \| \| ○ \| \| \| No \| \| \|  \| \| \| \| \|  \| \| \| \| \|  \| \| \|  \| \| \| \| iv \| Organizing help, physical aids or house adaptations? \| \| \| ○ \| \| \| Yes \| \| \| → \| \| \| \| \|  \| \| \| \| \| or \| \| \|  \| \|  \| \| ○ \| \| \| No \| \| \|  \| \| \| \| \|  \| \| \| \| \|  \| \| \|  \| \| \| \| v \| Taking care of financial matters like buying and selling things? \| \| \| ○ \| \| \| Yes \| \| \| → \| \| \| \| \|  \| \| \| \| \| or \| \| \|  \| \|  \| \| ○ \| \| \| No \| \| \|  \| \| \| \| \|  \|  \|  \| \| \| \| \| \| \| \| \| |
| --- | --- | --- | --- | --- | --- | --- | --- | --- | --- | --- | --- | --- | --- | --- | --- | --- | --- | --- | --- | --- | --- | --- | --- | --- | --- | --- | --- | --- | --- | --- | --- | --- | --- | --- | --- | --- | --- | --- | --- | --- | --- | --- | --- | --- | --- | --- | --- | --- | --- | --- | --- | --- | --- | --- | --- | --- | --- | --- | --- | --- | --- | --- | --- | --- | --- | --- | --- | --- | --- | --- | --- | --- | --- | --- | --- | --- | --- | --- | --- | --- | --- | --- | --- | --- | --- | --- | --- | --- | --- | --- | --- | --- | --- | --- | --- | --- | --- | --- | --- | --- | --- | --- | --- | --- | --- | --- | --- | --- | --- | --- | --- | --- | --- | --- | --- | --- | --- | --- | --- | --- | --- | --- | --- | --- | --- | --- | --- | --- | --- | --- | --- | --- | --- | --- | --- | --- | --- | --- | --- | --- | --- | --- | --- | --- | --- | --- | --- | --- | --- | --- | --- | --- | --- | --- | --- | --- | --- | --- | --- | --- | --- | --- | --- | --- | --- | --- | --- | --- | --- | --- | --- | --- | --- | --- | --- | --- | --- | --- | --- | --- | --- | --- | --- | --- | --- | --- | --- | --- | --- | --- | --- | --- | --- | --- | --- | --- | --- | --- | --- | --- | --- | --- | --- | --- | --- | --- | --- | --- | --- | --- | --- | --- | --- | --- | --- | --- | --- | --- | --- | --- | --- | --- | --- | --- | --- | --- | --- | --- | --- | --- | --- | --- | --- | --- | --- | --- | --- | --- | --- | --- | --- | --- | --- | --- | --- | --- | --- | --- | --- | --- | --- | --- | --- | --- | --- | --- | --- | --- | --- | --- | --- | --- | --- | --- | --- | --- | --- | --- | --- | --- | --- | --- | --- | --- | --- | --- | --- | --- | --- | --- | --- | --- | --- | --- | --- | --- | --- | --- | --- | --- | --- | --- | --- | --- | --- | --- | --- | --- | --- | --- | --- | --- | --- | --- | --- | --- | --- | --- | --- | --- | --- | --- | --- | --- | --- | --- | --- | --- | --- | --- | --- | --- | --- | --- | --- | --- | --- | --- | --- | --- | --- | --- | --- | --- | --- | --- | --- | --- | --- | --- | --- | --- | --- | --- | --- | --- | --- | --- | --- | --- | --- | --- | --- | --- | --- | --- | --- | --- | --- | --- | --- | --- | --- | --- | --- | --- | --- | --- | --- | --- | --- | --- | --- | --- | --- | --- | --- | --- | --- | --- | --- | --- | --- | --- | --- | --- | --- | --- | --- | --- | --- | --- | --- | --- | --- | --- | --- | --- | --- | --- | --- | --- | --- | --- | --- | --- | --- | --- | --- | --- | --- | --- | --- | --- | --- | --- | --- | --- | --- | --- | --- | --- | --- | --- | --- | --- | --- | --- | --- | --- | --- | --- | --- | --- | --- | --- | --- | --- | --- | --- | --- | --- | --- | --- | --- | --- | --- | --- | --- | --- | --- | --- | --- | --- | --- | --- | --- | --- | --- | --- | --- | --- | --- | --- | --- | --- | --- | --- | --- | --- | --- | --- | --- | --- | --- | --- | --- | --- | --- | --- | --- | --- | --- | --- | --- | --- | --- | --- | --- | --- | --- | --- | --- | --- | --- | --- | --- | --- | --- | --- | --- | --- | --- | --- | --- | --- | --- | --- | --- | --- | --- | --- | --- | --- | --- | --- | --- | --- | --- | --- | --- | --- | --- | --- | --- | --- | --- | --- | --- | --- | --- | --- | --- | --- | --- | --- | --- | --- | --- | --- | --- | --- | --- | --- | --- | --- | --- | --- | --- | --- | --- | --- | --- | --- | --- | --- | --- | --- | --- | --- | --- | --- | --- | --- | --- | --- | --- | --- | --- | --- | --- | --- | --- | --- | --- | --- | --- | --- | --- | --- | --- | --- | --- | --- | --- | --- | --- | --- | --- | --- | --- | --- | --- | --- | --- | --- | --- | --- | --- | --- | --- | --- | --- | --- | --- | --- | --- | --- | --- | --- | --- | --- | --- | --- | --- | --- | --- | --- | --- | --- | --- | --- | --- | --- | --- | --- | --- | --- | --- | --- | --- | --- | --- | --- | --- | --- | --- | --- | --- | --- | --- | --- | --- | --- | --- | --- | --- | --- | --- | --- | --- | --- | --- | --- | --- | --- | --- | --- | --- | --- | --- | --- | --- | --- | --- | --- | --- | --- | --- | --- | --- | --- | --- | --- | --- | --- | --- | --- | --- | --- | --- | --- | --- | --- | --- | --- | --- | --- | --- | --- | --- | --- | --- | --- | --- | --- | --- | --- | --- | --- | --- | --- | --- | --- | --- | --- | --- | --- | --- | --- | --- | --- | --- | --- | --- | --- | --- | --- | --- | --- | --- | --- | --- | --- | --- | --- | --- | --- | --- | --- | --- | --- | --- | --- | --- | --- | --- | --- | --- | --- | --- | --- | --- | --- | --- | --- | --- | --- | --- | --- | --- | --- | --- | --- | --- | --- | --- | --- | --- | --- | --- | --- | --- | --- | --- | --- | --- | --- | --- | --- | --- | --- | --- | --- | --- | --- | --- | --- | --- | --- | --- | --- | --- | --- | --- | --- | --- | --- | --- | --- | --- | --- | --- | --- | --- | --- | --- | --- | --- | --- | --- | --- | --- | --- | --- | --- | --- | --- | --- | --- | --- | --- | --- | --- | --- | --- | --- | --- | --- | --- | --- | --- | --- | --- | --- | --- | --- | --- | --- | --- | --- | --- | --- | --- | --- | --- | --- | --- | --- | --- | --- | --- | --- | --- | --- | --- | --- | --- | --- | --- | --- | --- | --- | --- | --- | --- | --- | --- | --- | --- | --- | --- | --- | --- | --- | --- | --- | --- | --- | --- | --- | --- | --- | --- | --- | --- | --- | --- | --- | --- | --- | --- | --- | --- | --- | --- | --- | --- | --- | --- | --- | --- | --- | --- | --- | --- | --- | --- | --- | --- | --- | --- | --- | --- | --- | --- | --- | --- | --- | --- | --- | --- | --- | --- | --- | --- | --- | --- | --- | --- | --- | --- | --- | --- | --- | --- | --- | --- | --- | --- | --- | --- | --- | --- | --- | --- | --- | --- | --- | --- | --- | --- | --- | --- | --- | --- | --- | --- | --- | --- | --- | --- | --- | --- | --- | --- | --- | --- | --- | --- | --- | --- | --- | --- | --- | --- | --- | --- | --- | --- | --- | --- | --- | --- | --- | --- | --- | --- | --- | --- | --- | --- | --- | --- | --- | --- | --- | --- | --- | --- | --- | --- | --- | --- | --- | --- | --- | --- | --- | --- | --- | --- | --- | --- | --- | --- | --- | --- | --- | --- | --- | --- | --- | --- | --- | --- | --- | --- | --- | --- | --- | --- | --- | --- | --- | --- |

| Q42.1 | Did you completely or partly give up paid work in order to provide informal care to her/him? | | | | |
| --- | --- | --- | --- | --- | --- |
|  | No, I did not have unpaid work before …………………..……………............ | | | 1 |  |
|  | No, I still perform the same amount of paid work …………………..…….. | | | 2 |  |
|  | Yes, | for ___________________(number) fewer hours per week since | | 3 |  |
|  |  | _____________________(year) |  |  |  |
|  |  | | | | |
|  |  | | | | |
| Q42.2 | Did you completely or partly give up unpaid work in order to provide informal care to her/him? | | | | |
|  | No, I did not have unpaid work before …………………..……………............ | | | 1 |  |
|  | No, I still perform the same amount of unpaid work …………………..…….. | | | 2 |  |
|  | Yes, | for ___________________(number) fewer hours per week since | | 3 |  |
|  |  | _____________________(year) |  |  |  |

|  |  | | | | |
| --- | --- | --- | --- | --- | --- |
| Q42.3 | Did you completely or partly give up time spent on leisure in order to provide informal care to her/him? | | | | |
|  | No, I did not have time for leisure before …………………..……………........ | | | 1 |  |
|  | No, I still perform the same amount of time on leisure …………………..… | | | 2 |  |
|  | Yes, | for ___________________(number) fewer hours per week since | | 3 |  |
|  |  | _____________________(year) |  |  |  |

| Q42.4 | Suppose you did not have to provide informal care anymore. How would you spend this time: on paid work, unpaid work, or leisure? | | | | | | | | | | | |
| --- | --- | --- | --- | --- | --- | --- | --- | --- | --- | --- | --- | --- |
|  |  | | | |  |  |  |  | | |  |  |
| i | - More paid work: | ○ | Yes | → | |  | | |  | Hours per week | | |
|  |  | ○ | No |  | |  | | |  |  | | |
| ii | - More unpaid work: | ○ | Yes | → | |  | | |  | Hours per week | | |
|  |  | ○ | No |  | |  | | |  |  | | |
| iii | - More leisure: | ○ | Yes | → | |  | | |  | Hours per week | | |
|  |  | ○ | No |  | |  | | |  |  | | |

| 43a): Section 5: | | *Zarit Burden Interview* | | | | | |
| --- | --- | --- | --- | --- | --- | --- | --- |
|  | The following is a list of statements which reflect how people sometimes feel when taking care of another person.  After each statement, indicate how often you feel that way: never, rarely, sometimes, quite frequently, or nearly always. There are no right or wrong answers. | | | | | | |
|  |  | | Never | Rarely | Sometimes | Quite frequently | Nearly always |
|  |  | | (0) | (1) | (2) | (3) | (4) |
| 1 | Do you feel that your relative asks for more help than he or she needs? | | ○ | ○ | ○ | ○ | ○ |
| 2 | Do you feel that, because of the time you spend with your relative, you don't have enough time for yourself? | | ○ | ○ | ○ | ○ | ○ |
| 3 | Do you feel stressed between caring for your relative and trying to meet other responsibilities for your family or work? | | ○ | ○ | ○ | ○ | ○ |
| 4 | Do you feel embarrassed about your relative's behavior? | | ○ | ○ | ○ | ○ | ○ |
| 5 | Do you feel angry when you are around your relative? | | ○ | ○ | ○ | ○ | ○ |
| 6 | Do you feel that your relative currently affects your relationship with other family members? | | ○ | ○ | ○ | ○ | ○ |
| 7 | Are you afraid about what the future holds for your relative? | | ○ | ○ | ○ | ○ | ○ |
| 8 | Do you feel that your relative is dependent upon you? | | ○ | ○ | ○ | ○ | ○ |
| 9 | Do you feel strained when you are around your relative? | | ○ | ○ | ○ | ○ | ○ |
| 10 | Do you feel that your health has suffered because of your involvement with your relative? | | ○ | ○ | ○ | ○ | ○ |
| 11 | Do you feel that you don't have as much privacy as you would like, because of your relative? | | ○ | ○ | ○ | ○ | ○ |
| 12 | Do you feel that your social life has suffered because you are caring for your relative? | | ○ | ○ | ○ | ○ | ○ |
| 13 | Do you feel uncomfortable having your friends over because of your relative? | | ○ | ○ | ○ | ○ | ○ |
| 14 | Do you feel that your relative seems to expect you to take care of him or her, as if you were the only one, he or she could depend on? | | ○ | ○ | ○ | ○ | ○ |
| 15 | Do you feel that you don't have enough money to care for your relative, in addition to the rest of your expenses? | | ○ | ○ | ○ | ○ | ○ |
| 16 | Do you feel that you will be unable to take care of your relative much longer? | | ○ | ○ | ○ | ○ | ○ |
| 17 | Do you feel that you have lost control of your life since your relative's illness? | | ○ | ○ | ○ | ○ | ○ |
| 18 | Do you wish that you could just leave the care of your relative to someone else? | | ○ | ○ | ○ | ○ | ○ |
| 19 | Do you feel uncertain about what to do about your relative? | | ○ | ○ | ○ | ○ | ○ |
| 20 | Do you feel that you should be doing more for your relative? | | ○ | ○ | ○ | ○ | ○ |
| 21 | Do you feel that you could do a better job in caring for your relative? | | ○ | ○ | ○ | ○ | ○ |
|  |  | | Not at all | A little | Moderately | Quite abit | Extremely |
|  |  | | (1) | (2) | (3) | (4) | (5) |
| 22 | Overall, how burdened do you feel in caring for your relative? | | ○ | ○ | ○ | ○ | ○ |

|  | 43b): Perceived financial burden  (*Economic cost component of the cost of care index*) | Strongly  Agree | Agree | Disagree | Strongly  Disagree |
| --- | --- | --- | --- | --- | --- |
|  |  | (1) | (2) | (3) | (4) |
| 1 | I feel that caring for my elderly relative is causing me (will cause me) to dip into savings meant for other things. | ○ | ○ | ○ | ○ |
| 2 | I feel that my family and I must give up (will have to give up) necessities because of the expense to care for my relative. | ○ | ○ | ○ | ○ |
| 3 | I feel that my family and I cannot (will not be able to) afford those little extras because of the expense to care for my relative. | ○ | ○ | ○ | ○ |
| 4 | I feel that caring for my relative is (will be) too expensive. | ○ | ○ | ○ | ○ |

| Q44 | | Respondents coping strategies [*16 item Coping Strategies Inventory Short Form (CSI-SF)]* | | | | | | |
| --- | --- | --- | --- | --- | --- | --- | --- | --- |
|  |  | This section is about how care givers cope with the various things involved in provision of informal care.  After each statement, please choose the number that best represents how often the statement describes your feelings [never, seldom, sometimes, almost always]. Again, there are no right or wrong answers. | | | | | | |
|  | | |  | Never | Seldom | Sometimes | Often | Almost always |
|  | | |  | (1) | (2) | (3) | (4) | (5) |
| PFE | 1 | | I make a plan of action and follow it | ○ | ○ | ○ | ○ | ○ |
|  | 2 | | I look for the silver lining or try to look on the bright side of things | ○ | ○ | ○ | ○ | ○ |
|  | 3 | | I tackle the problem head on | ○ | ○ | ○ | ○ | ○ |
|  | 4 | | I step back from the situation and try to put things into perspective | ○ | ○ | ○ | ○ | ○ |
| PFD | 5 | | I hope the problem will take care of itself | ○ | ○ | ○ | ○ | ○ |
|  | 6 | | I try to put the problem out of my mind | ○ | ○ | ○ | ○ | ○ |
|  | 7 | | I hope for a miracle | ○ | ○ | ○ | ○ | ○ |
|  | 8 | | I try not to think about the problem | ○ | ○ | ○ | ○ | ○ |
| EFD | 9 | | I try to let my emotions out | ○ | ○ | ○ | ○ | ○ |
|  | 10 | | I try to talk about it with a friend or family | ○ | ○ | ○ | ○ | ○ |
|  | 11 | | I let my feelings out to reduce the stress | ○ | ○ | ○ | ○ | ○ |
|  | 12 | | I ask a close friend or relative that I respect for help or advice | ○ | ○ | ○ | ○ | ○ |
| EFD | 13 | | I try to spend time alone | ○ | ○ | ○ | ○ | ○ |
|  | 14 | | I tend to blame myself | ○ | ○ | ○ | ○ | ○ |
|  | 15 | | I tend to criticize myself | ○ | ○ | ○ | ○ | ○ |
|  | 16 | | I keep my thoughts and feelings to myself | ○ | ○ | ○ | ○ | ○ |

| Q45 Section 6 | | ***Seizure frequency and severity*** | | |
| --- | --- | --- | --- | --- |
| Questions relate to your seizures or your child's seizures in the past 3 months. | | | | |
| a | In the last 12 months, has your child had a seizure lasting more than 5 minutes or back-to-back seizures? | | Yes 1  No 2  Unsure 3 |  |
| b | When was his/her last seizure? | | Today 1 |  |
|  |  |  | Up to I week ago 2 |  |
|  |  |  | Up to I month ago 3 |  |
|  |  |  | 3 - 6 months ago 4 |  |
|  |  |  | 3 - 6 months ago 5 |  |
|  |  |  | 6 - 12 months ago 6 |  |
|  |  |  | 13 - 24 months ago 7 |  |
|  |  |  | More than 2 years ago 8 |  |
|  |  |  | Unsure 9 |  |
| c | How frequent are they? | | Multiple per day 1 |  |
|  |  |  | Daily 2 |  |
|  |  |  | Weekly but not daily 3 |  |
|  |  |  | Monthly but not weekly 4 |  |
|  |  |  | At least one per year, but not every month…………………………………….5 |  |
|  |  |  | Less than once per year 6 |  |
|  |  |  | Unsure 7 |  |
|  |  | |  |  |
|  |  | |  |  |
|  | ***The Hague seizure severity (SS) scale*** | | |  |
|  | Questions relate to your seizures or your child's seizures in the past 3 months. | | |  |
| Q1. | How often do you notice a decrease of consciousness during a seizure in your child? | | Always 1 |  |
|  |  |  | Usually 2 |  |
|  |  |  | Sometimes 3 |  |
|  |  |  | Never 4 |  |
| Q2. | How long does such a decrease of consciousness last? (From time of onset to time of normal consciousness) | | Very long 1 |  |
|  |  |  | Long 2 |  |
|  |  |  | Short 3 |  |
|  |  |  | Very short 4 |  |
| Q3. | How severe have the seizures been overall? | | Very Severe 1 |  |
|  |  |  | Severe 2 |  |
|  |  |  | Mild 3 |  |
|  |  |  | Very mild 4 |  |
| Q4. | Are there any muscle jerks or cramps in the arms or legs during an attack? | | Always 1 |  |
|  |  |  | Usually 2 |  |
|  |  |  | Sometimes 3 |  |
|  |  |  | Never 4 |  |
| Q5. | How long do the jerks or cramps last during an attack? | | Very long 1 |  |
|  |  |  | Long 2 |  |
|  |  |  | Short 3 |  |
|  |  |  | Very short 4 |  |
|  |  |  | Does not apply, there are no jerks or cramps ...5 |  |

| Q.6 | How noticeable are the seizure symptoms? | Very noticeable, everyone will notice an attack 1 |  |
| --- | --- | --- | --- |
|  |  | Fairly noticeable, most people will notice an attack 2 |  |
|  |  | not very noticeable, most people will not notice 3 |  |
|  |  | not at all noticeable, you have to be very alert to notice an attack……………… 4 |  |
| Q7 | During or after an attack, how often does your child seem confused? | Always 1 |  |
|  |  | Usually 2 |  |
|  |  | Sometimes 3 |  |
|  |  | Never 4 |  |
| Q8 | During an attack, how often does your child wet him/herself? | Always 1 |  |
|  |  | Usually 2 |  |
|  |  | Sometimes 3 |  |
|  |  | Never or unknown, my child is permanently inconsistent .. 4 |  |
| Q9 | During an attack, how often does your child bite his/her tongue? | Always 1 |  |
|  |  | Usually 2 |  |
|  |  | Sometimes 3 |  |
|  |  | Never 4 |  |
| Q10 | How often does your child become injured during an attack (other than biting the tongue)? | Always 1 |  |
|  |  | Usually 2 |  |
|  |  | Sometimes 3 |  |
|  |  | Never 4 |  |
| Q11 | After the attack has finished, is your child sleepy? (including sleepiness caused by the use of rescue medication) | Always 1 |  |
|  |  | Usually 2 |  |
|  |  | Sometimes 3 |  |
|  |  | Never 4 |  |
| Q12 | After an attack, does your child complain of sickness, headache and/or pain in the muscles? | Always 1 |  |
|  |  | Usually 2 |  |
|  |  | Sometimes 3 |  |
|  |  | Never or unknown, my child would not be able to complain about that 4 |  |
| Q13 | After an attack, how long does it take, until your child can resume normal activity? | Very long 1 |  |
|  |  | Long 2 |  |
|  |  | Short 3 |  |
|  |  | Very short or direct after attack. 4 |  |
